# Double trouble: A case of fraternal twins with iron‐refractory iron‐deficiency anemia

**DOI:** 10.1002/ccr3.6401

**Published:** 2022-10-13

**Authors:** Jacques A. J. Malherbe, Catherine H. Cole

**Affiliations:** ^1^ Department of Haematology Fiona Stanley Hospital Murdoch Western Australia Australia; ^2^ School of Biomedical Sciences University of Western Australia Crawley Western Australia Australia; ^3^ Saturn Pathology Stirling Western Australia Australia

**Keywords:** anemia, hypoferritinemia, iron deficiency

## Abstract

Iron‐refractory iron‐deficiency anemia (IRIDA) is a rare autosomal recessive disease that presents in childhood. We report the case of fraternal twins presenting with severe hypochromic microcytic anemia and hypoferritinemia. Two missense mutations affecting the *TRMPSS6* gene were identified, consistent with IRIDA. Subsequent parenteral iron therapy improved clinical and blood parameters.

## 1 INTRODUCTION

Iron‐deficiency anemia in children is usually attributed to poor dietary intake of oral iron, malabsorption secondary to inhibitory substances (e.g., cow's milk) and chronic inflammatory conditions affecting the gastrointestinal tract (e.g., coeliac disease, *Strongyloides stercoralis*).^1^, ^2^, ^3^ Upon treatment of the underlying cause, most individuals respond to oral iron therapy with marked improvements in their clinical symptomatology and red cell indices.^3^ However, patients who are refractory to oral supplementation should provoke further investigation into uncommon and rare disorders affecting iron absorption, metabolism, and erythropoiesis (i.e., heme and globin manufacture). These disorders include thalassemia, sideroblastic anemia, and iron‐refractory iron‐deficiency anemia (IRIDA).^1^, ^2^

Iron‐refractory iron‐deficiency anemia is a rare, autosomal recessive disorder and usually presents in early childhood.^1^, ^2^ Such patients present with severe microcytic anemia, profound hypoferremia, low transferrin saturation, elevated hepcidin levels, and negligible responses to oral iron replacement therapy.^2^ The disorder arises from mutations affecting the *TMPRSS6* gene located on the long arm of chromosome 22q12.3.^2^, ^4^ The gene encodes matriptase‐2 (MT‐2), a transmembrane serine protease expressed by hepatocytes that once activated, downregulates hepcidin expression by interfering with hemojuvelin‐hepcidin coupling. This abrogates the inhibitory functions of hepcidin, thereby augmenting iron absorption from the gut and facilitating the mobilization of iron stores from macrophages.^2^, ^4^

Mutations affecting the *TMPRSS6* gene were first discovered in five multiplex kindreds by Finberg and colleagues in 2008.^4^ Since then, several missense, nonsense, frameshift, and splicing mutations presenting in compound heterozygote or single homozygote forms have been identified among an array of ethnicities and across all continents except Australasia.^5^, ^6^, ^7^, ^8^, ^9^, ^10^, ^11^, ^12^ Missense mutations affecting *TMPRSS6* are generally associated with less severe microcytic anemias and lower hepcidin levels in comparison with those IRIDA patients with other mutation types.^4^, ^6^, ^7^, ^12^ These patients also tend to show some clinical response to oral iron replacement therapy. IRIDA patients refractory to oral iron therapy require parental supplementation.^2^, ^6^ Newer agents targeting hepcidin signaling cascades have shown promising therapeutic benefits in murine models.^13^

We report IRIDA in fraternal twins who failed to respond to oral replacement therapy. Massively parallel gene sequencing of *TMPRSS6* confirmed the presence of two missense mutations expressed in a compound heterozygous genotype. The case highlights the genetic and clinical heterogeneity of patients with IRIDA despite common mutations affecting *TMPRSS6* and the occasional need for parenteral iron supplementation.

## 2 CASE PRESENTATION

A nine‐year‐old, non‐Indigenous, Caucasian Australian boy (twin #1) was referred by his general practitioner to the pediatric gastroenterology service at our State's children hospital for investigation and workup of his severe microcytic anemia that was not responding to a one‐month trial of oral 5 mg/kg of ferrous sulphate liquid (30 mg/mL) therapy. His weight, height, and growth were stable and normal for his age. His diet was varied and included regular red meat intake without excess cow's milk consumption. Despite the anemia (Hb 69 g/L), he was asymptomatic, and his mother reported no history of unexplained fevers or drenching night sweats. He had no personal or family history of chronic inflammatory disorders.

On examination, his vital signs were within the normal range and his oxygen saturation was >95% on room air. He exhibited marked pallor of the palmar creases, conjunctiva, and face. A comprehensive systems examination was otherwise unremarkable with no organomegaly or lymphadenopathy. Initial full blood count and serum iron studies (Table 1) showed a profound hypochromic microcytic anemia (Hb 69 g/L, MCV 56 fL), normal erythrocyte sedimentation rates (7.0 mm/hour), an increased red cell distribution width (22.0%), severe hypoferremia (<2.0 μmol/L) with very low transferrin saturations (0.03%) and normal serum ferritin levels (32.0 μg/L). Gastroenterological investigations were undertaken, including a gastroscopy, which elicited no evidence for *Helicobacter pylori* or *Strongyloides stercoralis* infections, coeliac disease, autoimmune gastritis, or evidence of other chronic inflammatory diseases. The patient was subsequently referred to the pediatric hematology service for further investigation and continued with oral iron replacement therapy.

**TABLE 1:** Hematological and biochemical parameters in both fraternal twins at diagnosis and 4 months post‐parenteral iron administration

Parameter	Pediatric reference range	Twin #1		Twin #2	
		Diagnosis	4 months post‐parenteral iron therapy	Diagnosis	4 months post‐parenteral iron therapy
WCC	4.50–14.50 × 10^9^/L	2.80	4.90	5.70	5.70
Platelets	150–400 × 10^9^/L	241	292	536	368
RCC	4.00–6.00 × 10^12^/L	4.1	5.26	5.17	5.59
Hb	115–155 g/L	61	94	76	100
Hct	0.35–0.45	0.26	0.32	0.27	0.33
MCV	75–92 fl	54	60	52	58
MCH	25.0–33.0 pg	15.0	18.0	14.6	17.8
MCHC	320–360 g/L	283	299	283	306
RDW	9.0%–15.0%	17.9	20.5	19.4	22.9
Iron	7–25 μmol/L	<2	4	3	4
Transferrin	20–41 μmol/L	35	27	35	30
Transferrin Saturation	12%–45%	0.03	7	4	7
Ferritin	15–100 μg/L	32	157	9	142

Abbreviations: fL, femtolitre; g, grams; Hb, hemoglobin; Hct, hematocrit; L, liter; MCH, mean cell hemoglobin; MCHC, mean cell hemoglobin concentration; MCV, mean cell volume; pg, picogram; RBC, red cell count; RDW, red cell distribution width; WCC, white cell count; μ, micro; %, percent.

**FIGURE 1:**
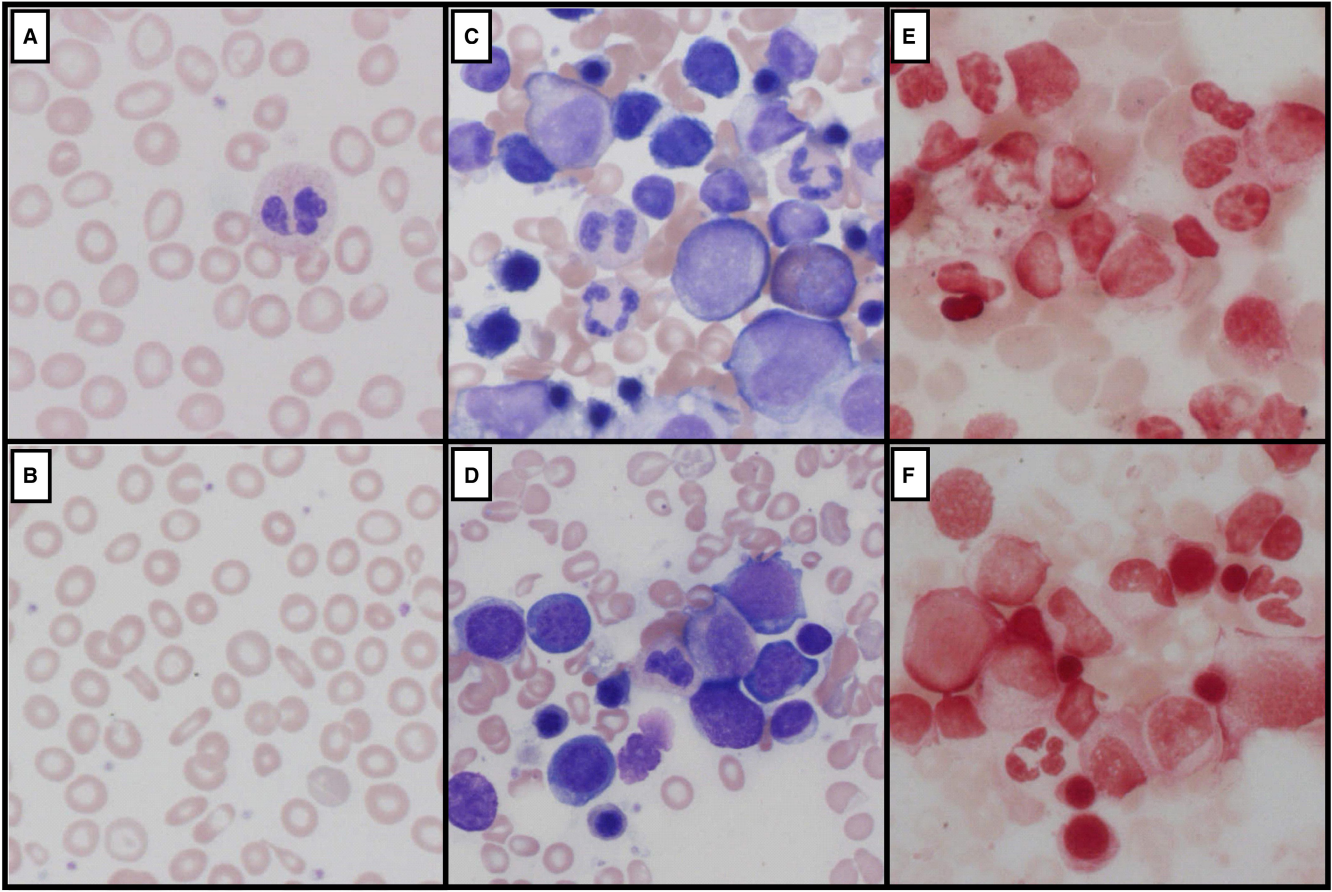
Representative blood films and bone marrow aspirate samples (600×) obtained from both twins prior to parenteral iron supplementation therapy. The upper and lower rows of images relate to twin #1 and twin #2, respectively. (A, B) Blood films from both twins show profound hypochromic erythrocytes with anisopoikilocytosis, occasional pencil cells and elliptocytes. (C, D) Bone marrow aspirate smears show normal trilineage hematopoiesis with corresponding (E, F) absent iron stores.

He was reviewed 5 months later in the outpatient hematology clinic alongside his fraternal twin brother (twin #2), who was noted to exhibit similar clinical features of central and peripheral pallor. Peripheral blood sampling of the second twin identified similar abnormalities to his brother (Table 1) and he was also commenced on oral iron replacement therapy. The blood films from both twins showed persisting moderate erythrocyte anisopoikilocytosis, pencil cells and elliptocytes despite ongoing oral iron therapy (Figure 1A,B) with negligible improvements in red cell indices, serum iron, and ferritin. Hemoglobin electrophoresis studies excluded a hemoglobinopathy in both siblings. Bone marrow biopsies were performed and showed normocellular trilineage hematopoiesis with no ringed sideroblasts or neoplasia. Occasional mild dyserythropoiesis characterized by nuclear budding, few binucleate forms and poor hemoglobinization were seen (Figure 1C,D). Cytogenetic analyses were normal although iron stores within the marrow were absent (Figure 1E,F). Hepcidin levels were not measured as an approved assay for this hormone was not available in our laboratory.

Given the ongoing poor response in both twins to oral iron replacement therapy, a possible diagnosis of IRIDA was considered. Massively parallel gene sequencing was performed on a peripheral blood sample of twin #1 one month later to assess for a *TMPRSS6* mutation. Two compound heterozygous c. (1324G > A; 1564G > A), p.(G442R; E522K) mutations were identified, confirming a diagnosis of IRIDA. No other novel mutations were identified in the *TMPRSS6* gene. Both twins were commenced on parenteral ferrous carboxymaltose 500 mg administered over 15 min 1 month later with concurrent cessation of oral iron therapy. This resulted in marked improvements in red cell indices and serum ferritin parameters. At 5 years following their initial diagnosis, both twins remain clinically well and have not required further parenteral iron supplementation.

## 3 DISCUSSION

We report IRIDA in fraternal twins who presented with severe microcytic anemia, hypoferremia, hypoferritinemia, and low transferrin saturation states. Two missense mutations (p. G442R; p. E522K) in the *TMPRSS6* gene presenting in a compound heterozygous form were detected using massively parallel gene sequencing. The mutations sequenced in our Australian twins have been previously identified in several other IRIDA patients.^4^, ^7^, ^12^ To date, only one other case report of a French‐Canadian kindred has identified the precise compound heterozygous missense mutations of *TMPRSS6* seen in our patients.^7^ In parallel with the findings reported by Khuong‐Quang et al. (2013),^7^ our patients presented with severe hypochromic microcytic anemia, hypoferremia, and negligible transferrin saturation. Similar blood count and serum iron abnormalities have been seen in other IRIDA patients with either the p.G442R or the p.E522K missense mutation.^4^, ^12^ However, unlike the French‐Canadian siblings,^7^ our twins showed borderline normal to low serum ferritin levels and failed to respond to simple oral iron replacement therapies. It was only after the administration of parenteral iron infusions that profound and sustained improvements in the hemoglobin and serum ferritin of our patients were observed.

Reasons for these clinical differences despite a similar genotype has been a contentious issue within the IRIDA arena. Genotype–phenotype studies of a large European cohort of IRIDA patients across multiple unrelated families have proposed that missense mutations affecting the *TMPRSS6* mutation through a compound heterozygous phenomenon typically present with less severe anemia and milder hypoferremia.^6^ In addition, they usually respond to oral iron therapy in comparison to patients with single homozygous mutations or other lesion types (e.g., frameshift, nonsense). However, this appears to be at odds with our patients. The clinical heterogeneity of IRIDA been attributed to diverse *TMPRSS6* mutations affecting variable domains within the encoded MT‐2 product, leading to different degrees of dysfunction.^6^, ^12^ The p.G442R mutation of the *TMPRSS6* gene is known to target and alter the activity of the second C1r/C1s, urchin embryonic growth factor and bone morphogenetic protein 1 (CUB) domain of MT‐2, which is involved in its autoactivation through autocatalytic cleavage.^4^, ^6^, ^12^ In contrast, the p.E522K mutation disrupts the second‐class A low‐density lipoprotein receptor (LDLRA) domain further downstream.^6^, ^12^ This domain has been proposed to assist with the conformational folding of MT‐2 to achieve autocatalytic cleavage, while also facilitating interactions between hemojuvelin and MT‐2. Silvestri and co‐workers (2009)^12^ showed through a series of separately transfected HeLa cell lines with multiple missense mutations targeting the *TMPRSS6* gene, including p.G442R and p.E522K, that the latter was more mutagenic. LDLRA domain alterations propagated through the p.E522K lesion showed reduced expression of MT‐2 on the cell membrane of transfected cells and was unable to interfere with hemojuvelin.^12^ In contrast, the p.G442R mutation and subsequent aberrations in the CUB domain did not alter the expression of the MT‐2 protein on the cell membrane.^12^ Moreover, CUB mutations still retained partial, albeit lowered hemojuvelin cleavage capabilities.^12^ A compound heterozygous inheritance mechanism for both missense mutations may therefore cause a severe IRIDA phenotype that does not respond to oral iron therapy through complete dysfunction of the mutated MT‐2 encoded product.

Of interest, we observed that despite a reasonable trial of oral iron replacement therapy, neither child showed any clinical improvement in red cell indices or serum iron levels. Previously published reports of IRIDA patients with missense mutations affecting the CUB and LDLRA domains, including the French‐Canadian kindred, have successfully treated their patients with oral iron supplements.^6^, ^7^ Interestingly, the French‐Canadian siblings only showed clinical improvements after 1 year of oral iron therapy in combination with ascorbic acid.^7^ The shorter time course and exclusive use of oral iron supplementation without ascorbic acid may account for the lack of response seen in our patients. Nevertheless, parenteral iron infusions are reported to yield clinical benefits. It has been previously shown that recovery of red cell indices and ferritin is often slow and prolonged with an ongoing need for parenteral iron administration.^2^, ^6^ Surprisingly, we observed marked and sustained improvements in red cells indices and ferritin levels in our patients after a single iron infusion.

In summary, we have reported IRIDA in pediatric, fraternal twins with compound heterozygous missense mutations in the *TMPRSS6* gene. We show that despite knowledge of these *TMPRSS6* mutations,^4^, ^6^, ^7^, ^12^ they confer extensive clinical heterogeneity. Therapy should therefore be directed on a case‐by‐case basis. Currently, the frequency of parenteral iron administration in IRIDA cases refractory to oral iron replacement therapy is not established. Future prospective cohort studies will hopefully elicit appropriate therapeutic guidelines for IRIDA.

## AUTHOR CONTRIBUTIONS

CHC cared for the patient. All authors collected, analyzed, and synthesized all longitudinal data. JAJM undertook a comprehensive literature review. JAJM wrote the manuscript. All authors critically revised and approved the final manuscript.

## FUNDING INFORMATION

No funding or financial support was received for the study.

## CONFLICT OF INTEREST

The authors declare that they have no conflicts of interest.

## CONSENT

Written informed consent for the publication of this case and the accompanying images was obtained from the patients' legal guardian.

## Data Availability

All data generated and pertaining to this case report are included in the manuscript.
